# Supplementary material for: Observation of 1D Fermi arc states in Weyl semimetal TaAs
Source: Natl Sci Rev. 2021 Oct 25;9(8):nwab191. doi: 10.1093/nsr/nwab191 (PMC9466953; doi:10.1093/nsr/nwab191)
Supplement: nwab191_Supplemental_File [file nwab191_supplemental_file.docx]

Supplementary Materials for

**Observation of 1D Fermi arc states in Weyl semimetal TaAs**

Xiaohu Zheng^1,2†^, Qiangqiang Gu^1†^, Yiyuan Liu^1^, Bingbing Tong^1,2^, Jian-Feng Zhang^1^, Chi Zhang^3^, Shuang Jia^1^, Ji Feng^1,4,5*^, Rui-Rui Du^1,4,5*^

^1^International Center for Quantum Materials, School of Physics, Peking University, Beijing 100871, China.

^2^Beijing Academy of Quantum Information Sciences, Beijing 100193, China.

^3^State Key Laboratory of Superlattices and Microstructures, Institute of Semiconductors, Chinese Academy of Sciences, Beijing 100083, China.

^4^CAS Center for Excellence in Topological Quantum Computation, University of Chinese Academy of Sciences, Beijing 100190, China.

^5^Collaborative Innovation Center of Quantum Matter, Beijing 100871, China.

† These authors contributed equally to this work.

*Correspondence to: [rrd@pku.edu.cn](mailto:rrd@rice.edu); [jfeng11@pku.edu.cn](mailto:jfeng11@pku.edu.cn).

**This PDF file includes:**

Fig. S1. Fermi arc states on (001) surface of TaAs.

Fig. S2. Step edge states on (001) surface of TaAs.

Fig. S3. Difference between processed and cleaved (112) surfaces.

Fig. S4. Construction of the calculation slab models with (110) terraced planes and atomic-thick (001) exposed at the step ledges.

Fig. S5. All visible Fermi arcs on the top and bottom surface with (110) terraces.

Fig. S6. Calculation of Fermi arc projection on (110) terraced surface as a function of energy.

Fig. S7. Construction of the calculation slab model with (112) terraced planes and atomic-thick (001) exposed at the step ledge.

Fig. S8. Surface states on the bottom surface with (112) terraces.

Fig. S9. Construction of the calculation slab model with (114) terraced planes and atomic-thick (001) exposed at the step ledge.

Fig. S10. 2D Fermi arc surface states on (114) terraced planes.

**Surface and edge states on Fermi arc-allowed-surface (001) in TaAs crystal**

The Fermi arc surface states distribute on the top and bottom surfaces of (001) crystal face in TaAs (as shown in Fig. S1A) owing to the broken inversion symmetry. Here, we have calculated the Fermi surfaces on As-terminated (001) facet, which contains the Fermi arcs, the trivial surface states and the bulk derived surface resonance states. By projecting the Fermi surface onto each of Ta- layers in the unit cell, we can see the trivial surface states decaying very fast, and survive prominently on the topmost surface. In contrast, the Fermi arcs can be observed on Ta- layers all over the unit cell. It demonstrates that Fermi arc surface states can disperse into the bulk with a particular depth in real space, which is comparable to the unit constant along $\vec{c}$direction (~1.2 nm).

We performed the STM/STS measurements on the *in-situ* cleaved fresh (001) surface. The atomic resolved topographic image is presented in Fig. S1C, which contains high density point defects (vacancies). The defects induced the electrons scattering inter/intra the Fermi contours, and forming the quasiparticle interference (QPI) patterns on the STS mapping (acquired with the sample bias voltage of -25 mV), as shown in Fig. S1d. By Fourier transform, the scattering wave vector can be observed in the momentum space. Fermi arcs and their connection to the bulk continuum can be detected by the QPI patterns. Our results on (001) are in accordance with the previous works, which demonstrate the high quality of the TaAs crystals.

On the cleaved (001) surface, we investigated the states at the step edge. As shown in Fig. S2A, there is a step with a minimal height. The LDOS has been measured by STS, which shows no peaked DOS at the step edge, as shown in Fig. S2B. By carefully comparing the spectra at the surface with those near the step edge, it is clearly shown that the LDOS are perturbed locally at step edge (Fig. S2C). In the *dI/dV* mappings acquired at various of bias voltages near the Fermi level (Fig. S2D), interference patterns originate from the electronic standing waves on the surface and near the step can be observed, but again, no prominent and uniform edge states can be detected.


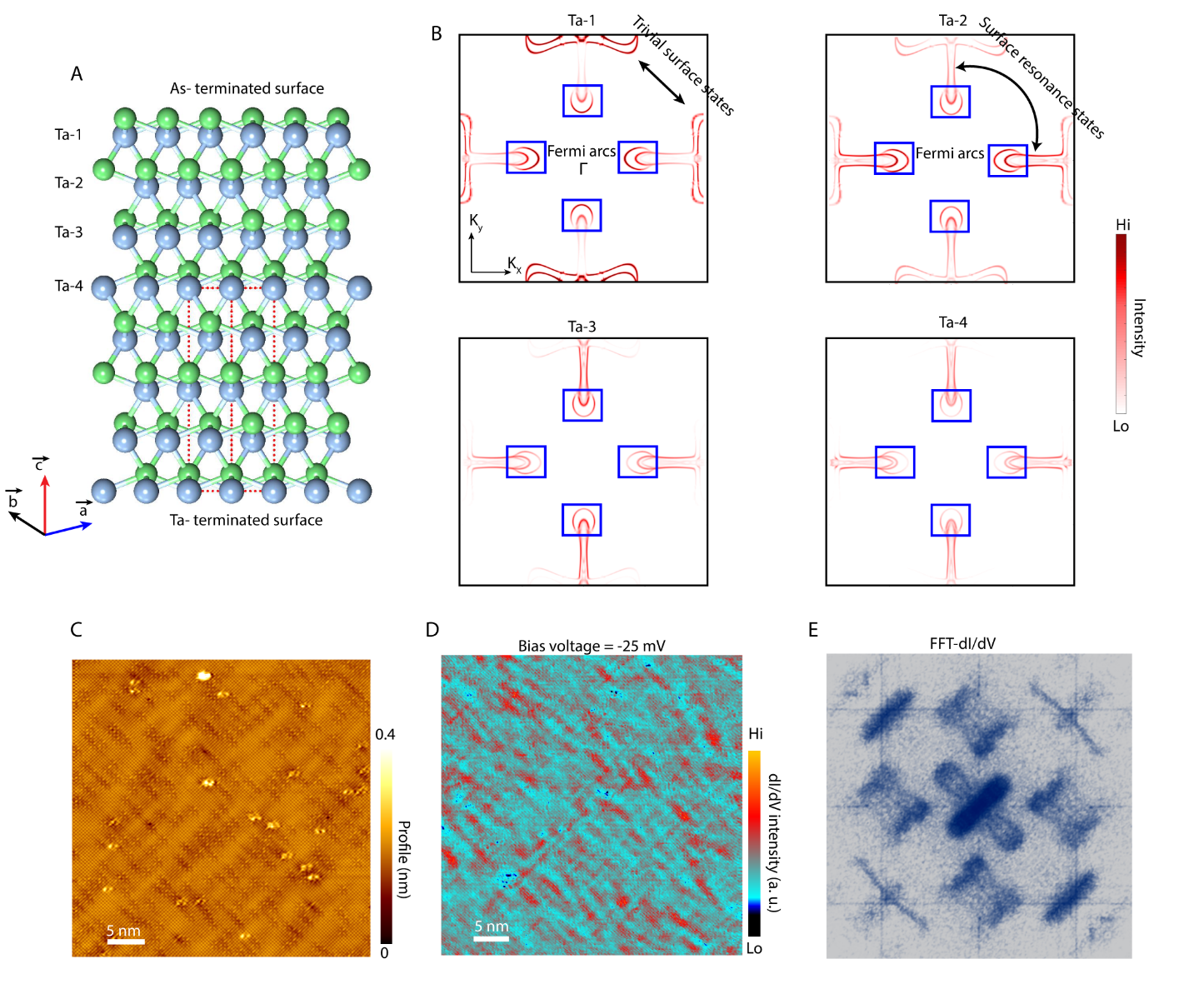


**Fig. S1. Fermi arc states on (001) surface of TaAs.** (**A**) Schematically shows the crystal structure of the TaAs crystal with As-termination on the top (001) face. There are four Ta- layers in a unit cell along $c$ direction (Ta-1, Ta-2, Ta-3 and Ta-4), the lattice constant $c$ is about 1.2 nm; (**B**) The projection of the calculated surface Fermi contours on each Ta- layer; (**C**) The topographic image of the cleaved (001) face with vacancy defects; (**D**) *dI/dV* conduction map at the region of (C) demonstrates the quasiparticle interference of electrons scattering around the defects; (**E**) Fourier transform of the conduction map (D) presents the scattering wave vectors in the momentum space.


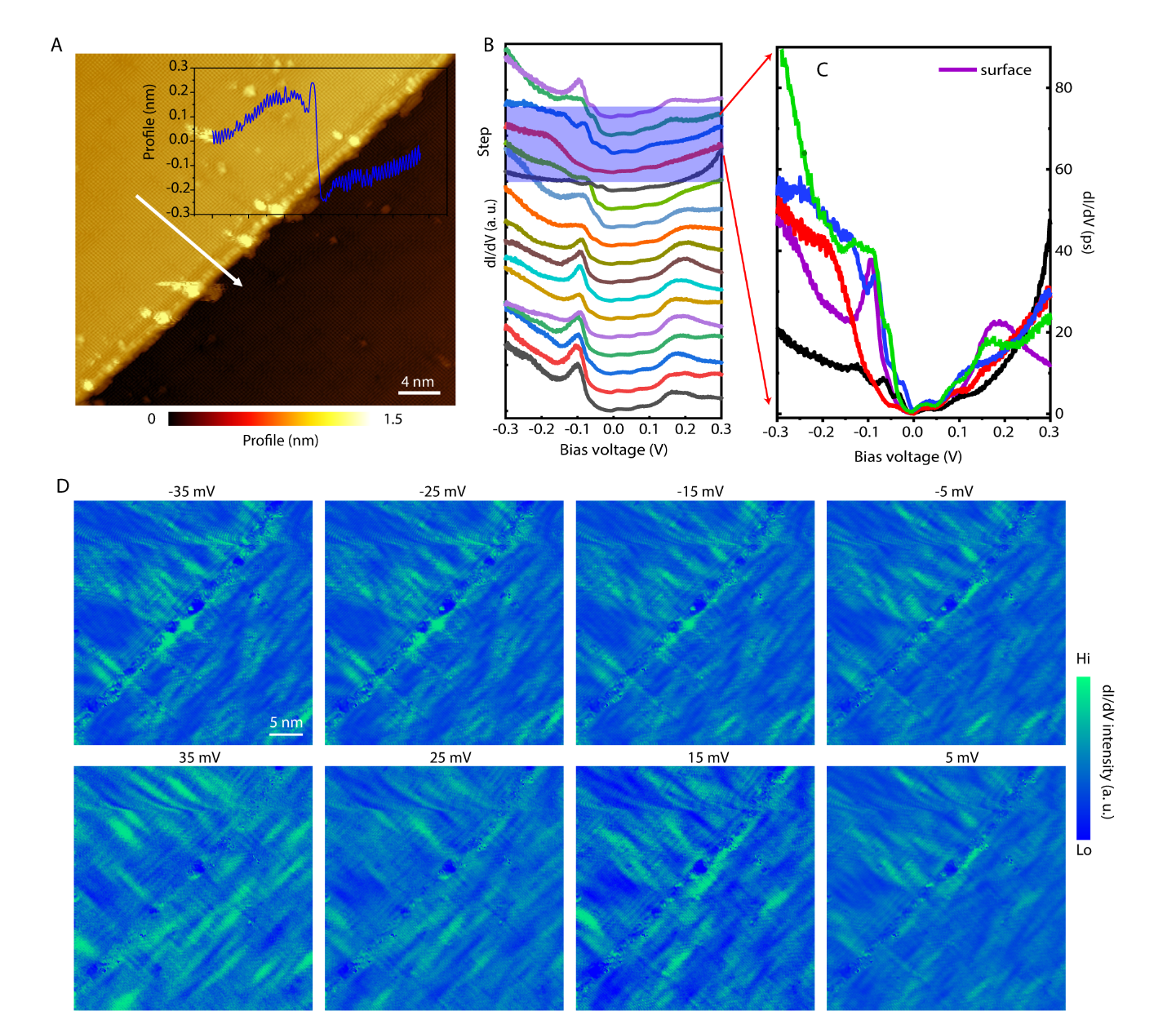


**Fig. S2. Step edge states on (001) surface of TaAs.** (**A**) Topographic image of the (001) face with a step edge. Inset shows the height profile of the step (~0.35 nm); (**B**) The tunneling spectra acquired across the step; (**C**) Near the step edge, the LDOS show position- and energy- dependence where no steadily peaked DOS can be observed; (**D**) *dI/dV* conduction mappings acquired at energy near the Fermi level from the region of (A) show the QPI patterns on the surface, but also no obvious steady and uniform 1D edge states can be observed.

**Surface relaxation in the processed (112) facet**

TaAs is a non-centrosymmetric transition-metal compound with a somewhat complex crystal structure. Cleavage is the method of choice to obtain the desired crystal face although it is particularly challenging for TaAs. In this work, we also develop a procedure with Ar^+^ bombardment and post-annealing, and obtained the terraced surface as presented in Fig. S3C. However, regardless of nominally the same processing conditions we have observed two types of surface atomic configurations on (112) facet in cleaved and processed samples. Fig. S3A and B show topographic and tunneling current panels acquired simultaneously from the same region of the cleaved (112) surface. The topographic image in Fig. S3A shows a perfect consistency with the Ta- arrangement in the structure model on (112) plane. It indicates that the surface relaxation is negligible during the cleave process. We note however, as revealed by the tunneling signal in Fig. S3B the atomic configurations show certain departure from the structural model. During the measurements, the STM is working in a constant current mode in acquiring a morphological image, there is a loop response time to hold the setpoint, the change of the tunneling current signals can be recorded, simultaneously. The panel in Fig. S3B reveals the modulation of tunneling current signals in each pixel. It contains negligible height information. Tunneling signals contributed from atoms in both the topmost and the second layers can be revealed. The deviation between (A) and (B) implies a strong hybridization of LDOS between the topmost and the underneath atoms. Fig. S3D shows the topographic STM image of the processed (112) surface. Here, the atom configuration is consistent with the tunneling signal image of the cleaved (112) surface in tunneling panel Fig. S3B. It indicates that the annealing process results in the relaxation of the surface atoms to achieve a more stable state.


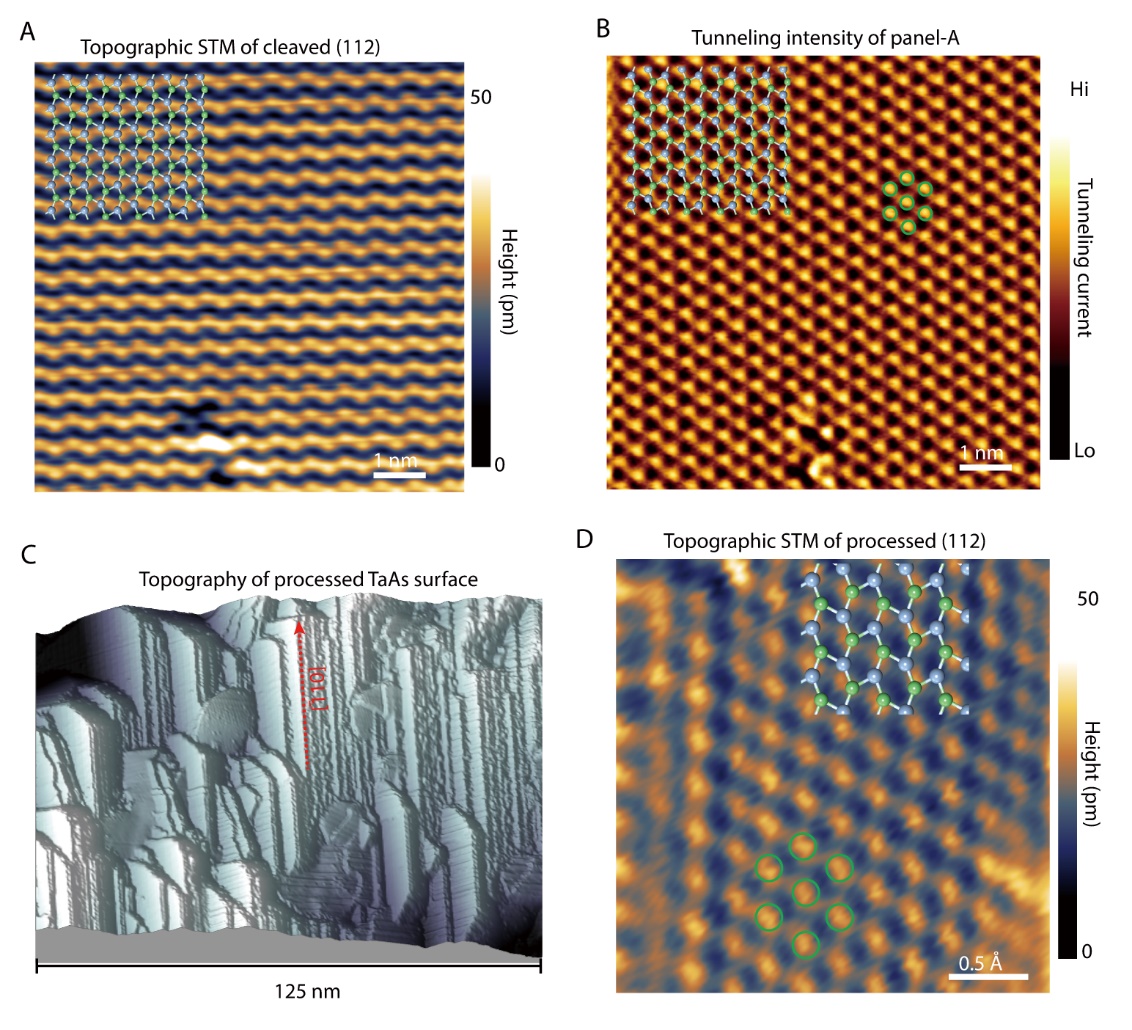


**Fig. S3. Difference between processed and cleaved (112) surfaces**. (**A**) and (**B**) Topographic and tunneling current panels acquired simultaneously from the same region of the cleaved (112) surface; (**C**) The large view topographic image acquired from the processed TaAs sample, where the steps along [$\bar{1}$10] can be observed; (**D**) Atomic resolved STM image acquired from the processed (112) facet.

**Calculations on terraced (110), (112) and (114) surface planes**


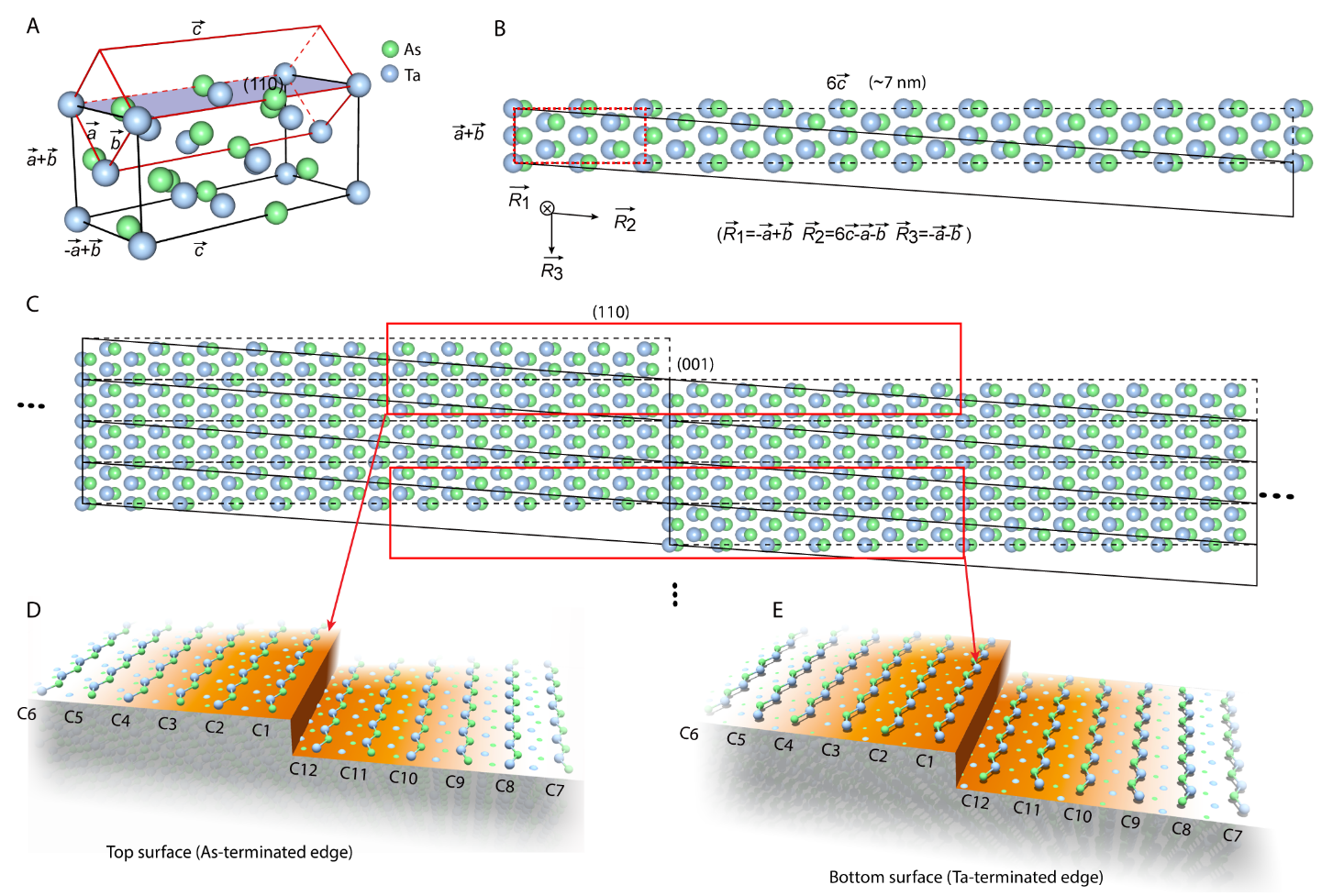


**Fig. S4. Construction of the calculation slab models with (110) terraced planes and atomic-thick (001) exposed at the step ledges.** (**A**) Transformation of TaAs crystal unit cell to a supercell with three lattice vectors $-\vec{a}+\vec{b}$**,** $\vec{a}+\vec{b}$ and $\vec{c}$, where $\vec{a}$, $\vec{b}$ and $\vec{c}$ are lattice vectors of the TaAs unit cell. The (110) plane is spanned by vectors $-\vec{a}+\vec{b}$ and $\vec{c}$. The black and red boxes represent the supercell and TaAs unit cell, respectively; (**B**) Construction of a larger supercell based on the supercell in (A), it is illustrated by the black solid rhomboid where half of the atoms in the supercell are selected outside the supercell lattice according to the periodic condition. Its lattice vectors are $\vec{R}_{1}=-\vec{a}+\vec{b}$, $\vec{R}_{2}=6\vec{c}-\vec{a}-\vec{b}$, $\vec{R}_{3}=-\vec{a}-\vec{b}$. $\vec{R}_{3}$ is the iterative direction, and $\vec{R}_{1}$, $\vec{R}_{2}$ define the surface plane $(n, n, 2)$ with $n=6$ where the surface states are calculated; (**C**) The schematic illustration of semi-infinite geometry formed in iterative Green’s function procedure based on the supercell in (B). Due to the specific atom occupation condition, the periodic step edges jointing (110) and (001) planes are generated automatically. In the calculation slab model, there are two different terraced surfaces (top and bottom), on the top surface **(D)**, the step ledges are terminated by As- atoms (constructed by small (001) with As-termination); and on the bottom surface (**E**), the step ledges are terminated by Ta- atoms (constructed by small (001) with Ta-termination). Ta-As chains labeled from C1 to C12 on each (110) terrace can be observed on the top (D) and the bottom (E) surfaces.


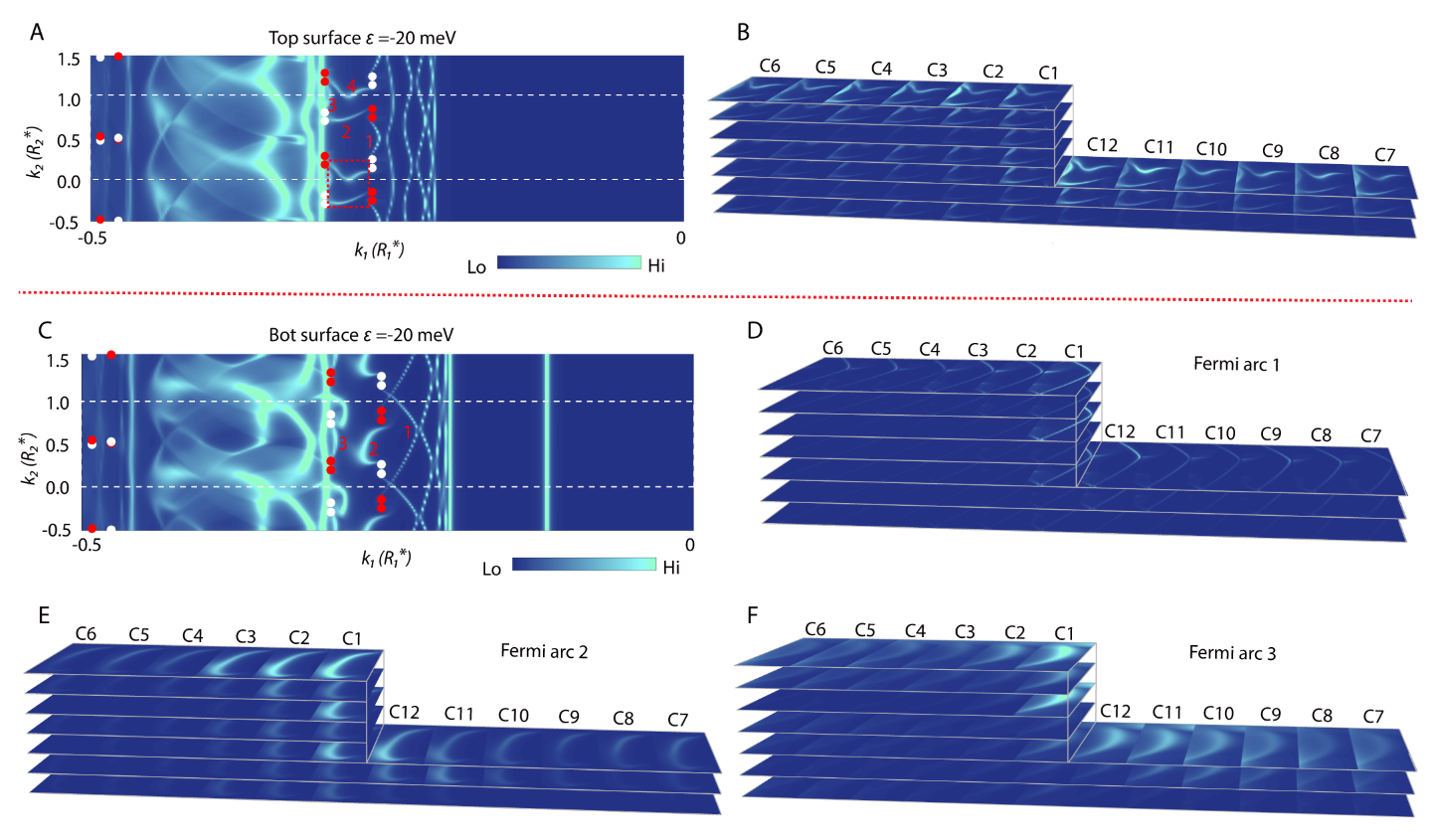


**Fig. S5. All visible Fermi arcs on the top and bottom surface with (110) terraces.** (**A**) Surface FS plots on the surface BZ (half of the whole BZ in the white dashed square) at $\varepsilon$= -20 meV with respect to bulk Fermi energy calculated based on the slab model in Fig. S4D (top surface). FSs on the whole surface BZ can be inferred through time-reversal symmetry. Near the center of the half BZ, there are 4 pairs of chiral Weyl points, and 4 Fermi arcs can be distinguished as numbered from 1 to 4. In the main text arc 1 is presented. Here, other three arcs that projected on the Ta-As chains on (110) terrace have been calculated. Distribution of the projection weight has been shown in (**B**), where the prominent projection weight can all be observed near the step and it decreases steadily as the position moving away from the step, in particular for the direction along (C12 to C7). (**C**) Surface FS plots on the surface BZ (half of the whole BZ in the white dashed square) at $\varepsilon$= -20 meV with respect to bulk Fermi energy calculated based on the slab model in Fig. S4E (bottom surface). There are three arcs that can be identified as numbered as 1, 2 and 3 near the center of the half BZ;(**D**) to (**E**) show the projections of the selected Fermi arc (1, 2 and 3) on the Ta-As chain as numbered in Fig. S4E. The prominent projection weights of the three Fermi arcs are all localized on Ta-As chains near the step edge and decrease steadily as the position far from the step which is in accordance with the calculation results on the top surface and can be well account for the 1D edge states at the step on (110) surface.


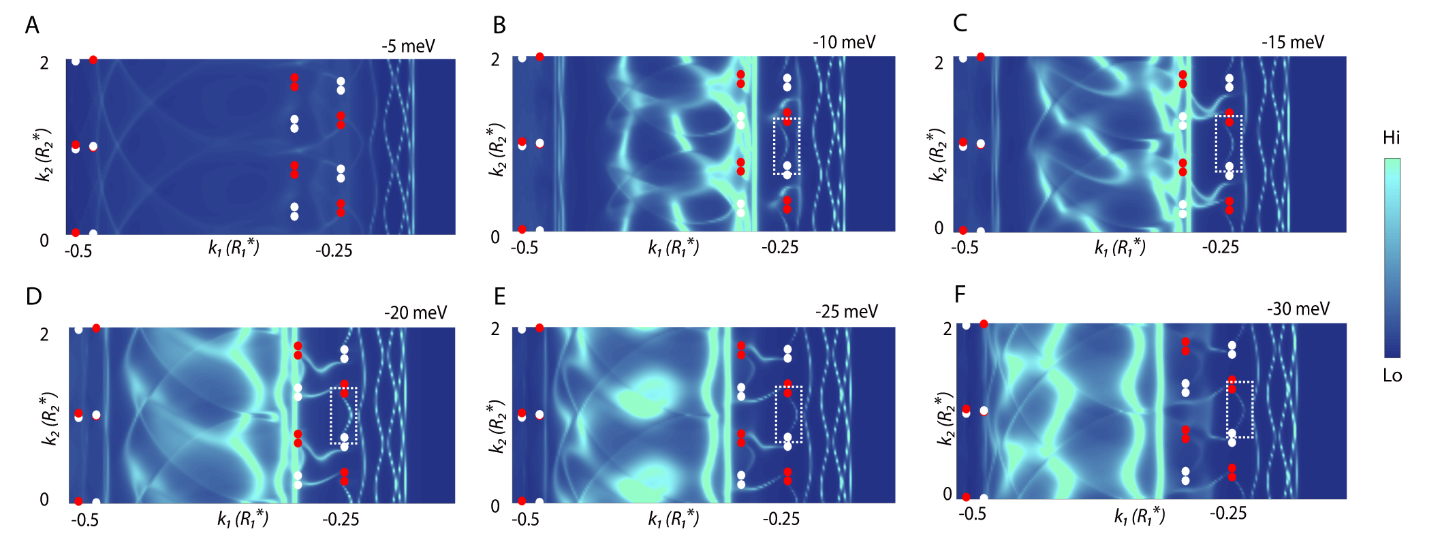


**Fig. S6. Calculation of Fermi arc projection on (110) terraced surface as a function of energy.** (**A**) to (**F**) Surface states on the top surface with (110) terraces at energy -5, -10, -15, -20, -25, -30 meV. The topological Fermi arc states exist near the Fermi level within the energy range between -5 ~ -30 meV.


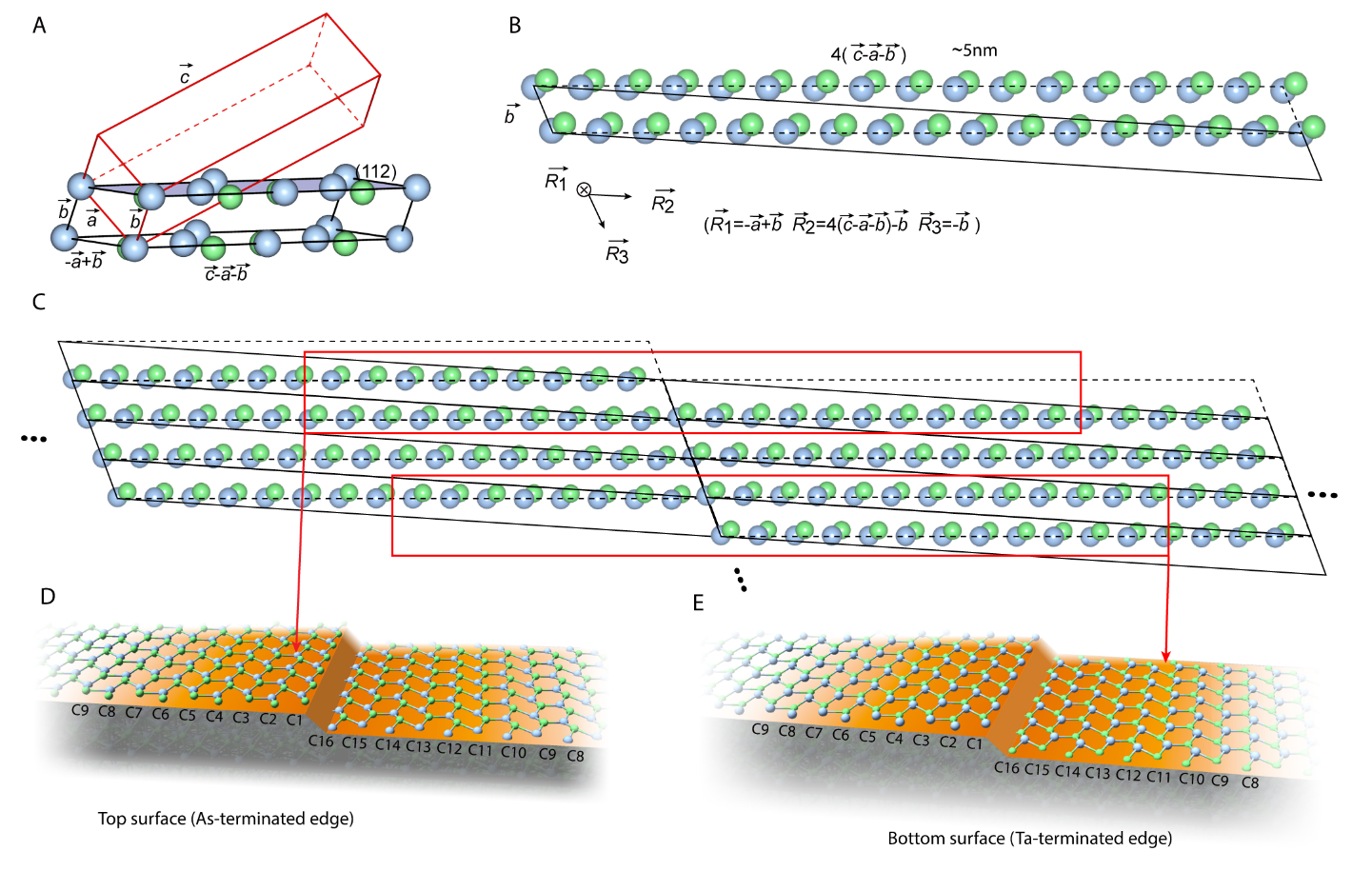


**Fig. S7. Construction of the calculation slab model with (112) terraced planes and atomic-thick (001) exposed at the step ledge.** (**A**) TaAs supercell with the lattice vectors $-\vec{a}+\vec{b}$, $\vec{b}$ and $\vec{c}-\vec{a}-\vec{b}$. The (112) plane is spanned by the supercell lattice vectors $-\vec{a}+\vec{b}$ and $\vec{c}-\vec{a}-\vec{b}$; (**B**) Construction of calculation model for (112) surface planes with periodic steps based on the supercell in (A). The iterative direction $\vec{R}_{3}$ in the iteration process is $-\vec{b}$. The two vectors $\vec{R}_{1}=-\vec{a}+\vec{b}$ and $\vec{R}_{2}=4\left( \vec{c}-\vec{a}-\vec{b} \right)-\vec{b}$ form the surface plane $(n, n, 2n+1)$with $n=4$ where the periodic step edges jointing (112) and (001) planes are formed automatically and the surface states are projected on; (**C**) The schematic illustration of semi-infinite geometry formed in iterative Green’s function procedure based on the supercell in (B). Due to the specific atom occupation condition, the periodic step edges jointing (112) and (001) planes are generated automatically. Two different terraced surfaces are formed meaning the top (**D**) and the bottom (**E**) with the step ledges terminated by As- and Ta- atoms respectively. Each (112) terrace has 16 Ta-As chains on the surface, as labeled from C1 to C16.


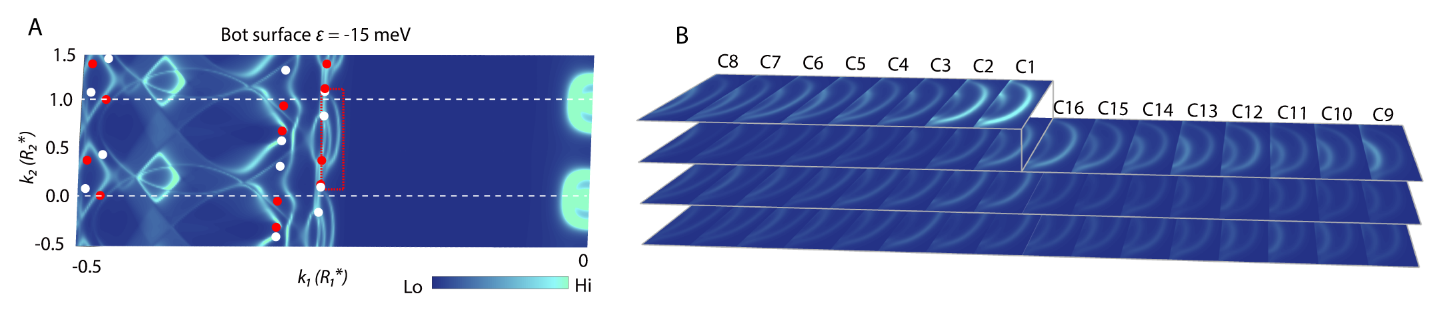


**Fig. S8. Surface states on the bottom surface with (112) terraces.** (**A**) Surface FS plots on half the surface BZ (white dashed square) at $\varepsilon$=-15 meV with respect to bulk Fermi energy calculated based on the slab model in Fig. S7E (bottom surface). The Fermi arc that is clearly isolated from trivial states is selected to calculate the projection weight on Ta-As chains as numbered in Fig. S7E. The projection weight is most prominent at Ta-As chains near the step edge and decreases slowly as the distance from the step increased as shown in (**B**), which is consistent with that on the top surface, as presented in the main text.


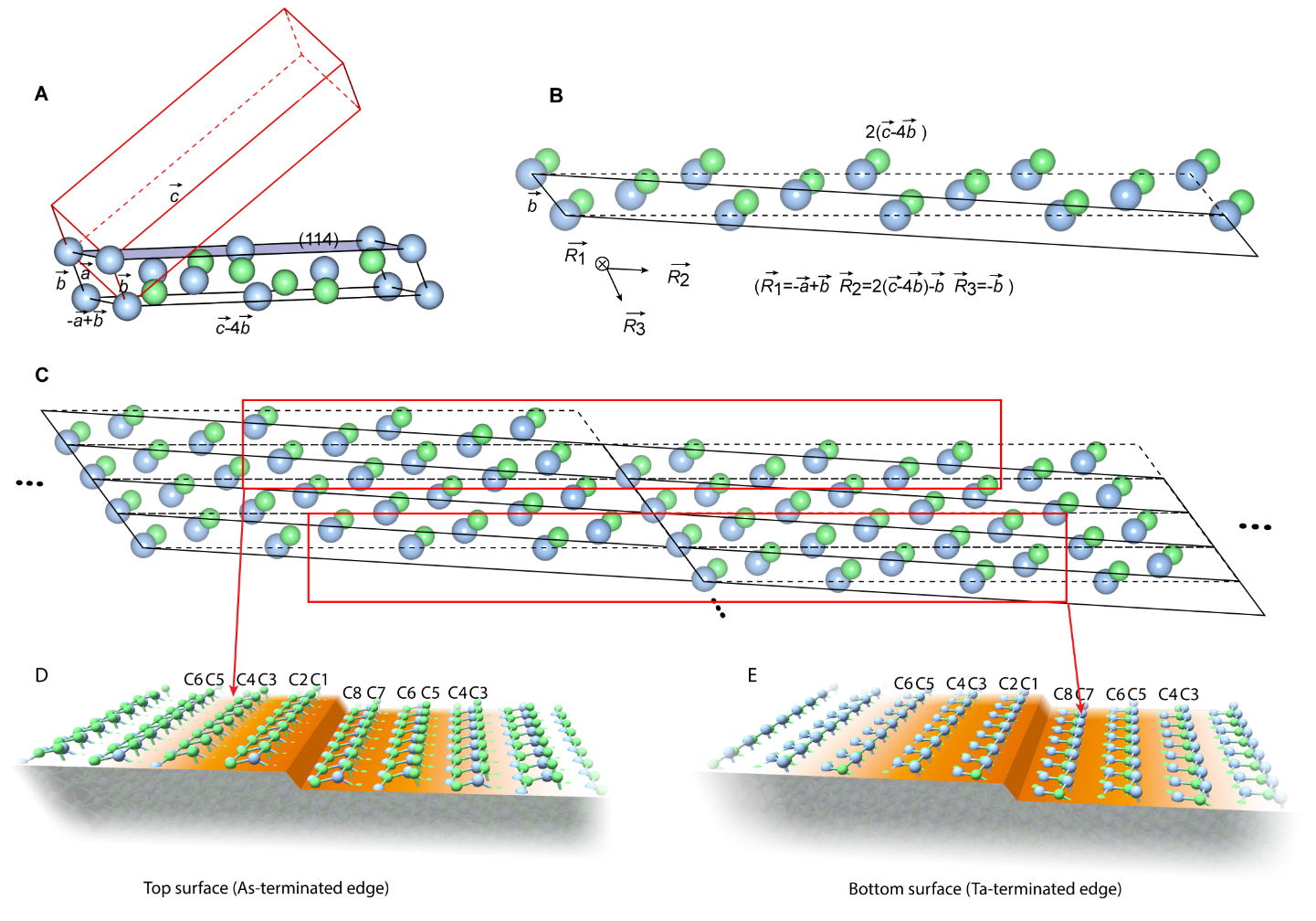


**Fig. S9. Construction of the calculation slab model with (114) terraced planes and atomic-thick (001) exposed at the step ledge.** (**A**) TaAs supercell with the lattice vectors $-\vec{a}+\vec{b}$**,**$\vec{b}$ and $\vec{c}-4\vec{b}$; (**B**) Construction of the calculation model for (114) surface.$\vec{R}_{1}$, $\vec{R}_{2}$ and $\vec{R}_{3}$ used in the iterative Green’s function scheme are defined as $\vec{R}_{1}=-\vec{a}+\vec{b}$, $\vec{R}_{2}=2(\vec{c}-4\vec{b})-\vec{b}$ and $\vec{R}_{3}=\vec{b}$.$\vec{R}_{3}$ is the iterative direction. Here, periodic steps jointing (114) and (001) are formed on the surface plane$(n, n, 4n+1) (n=2)$ spanned by vectors $\vec{R}_{1}$ and $\vec{R}_{2}$; (**C**) The schematic illustration of semi-infinite slab formed in iterative Green’s function procedure based on the supercell in (B). Periodic step edges jointing (114) and (001) planes are generated on the (229) surface with flat (114) terrace in the slab with As- terminated edge on the top surface (**D**)**,** and Ta- terminated edge on the bottom surface (**E**). Each terrace has 8 Ta-As chains surface as labeled from C1 to C8.


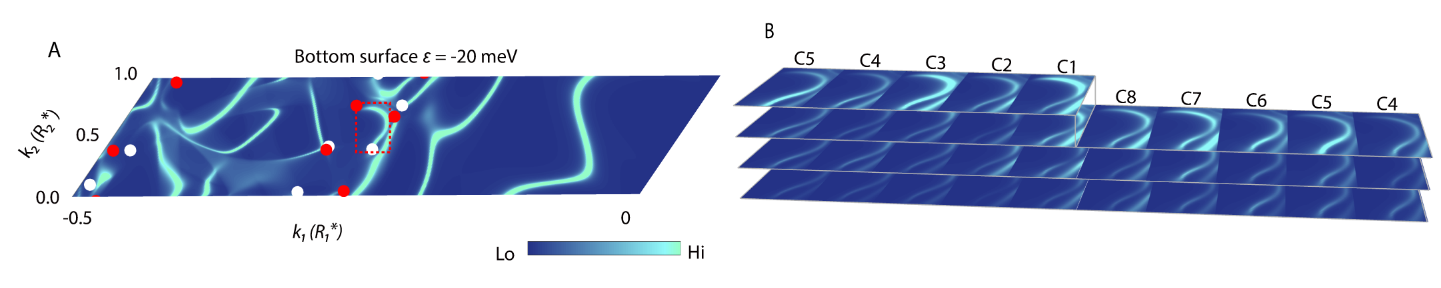


**Fig. S10. 2D Fermi arc surface states on (114) terraced planes.** Surface Fermi surface (FS) plots on **(A)** bottom surface at $\varepsilon=$-20 meV have been drawn in half the surface BZ. The bottom surface has been described in Fig. S9(E). The Fermi arcs that connecting chiral Weyl points and being isolated from trivial states have been demonstrated; **(B)** show the projection weight distribution of selected Fermi arcs on the bottom surfaces. The arcs can be observed on each of Ta-As chains on the topmost surfaces without significantly enhancing at the step edges on the bottom surfaces, which is consistent with the experimental results that no obvious edge state has been detected. The results confirm there are prominent Fermi arc surface states on (114) crystal facets.
